# PLA2G7: a new player in shaping energy metabolism and lifespan

**DOI:** 10.1038/s41392-022-01052-5

**Published:** 2022-06-17

**Authors:** Lena Susanna Candels, Svea Becker, Christian Trautwein

**Affiliations:** Department of Medicine III, University Hospital RWTH Aachen, Aachen, Germany

**Keywords:** Biomarkers, Prognostic markers

In a recent study published in *science*, Spadaro and colleagues described the downregulation of *PLA2G7* through sustained calory restriction as a crucial factor for lowering inflammatory mechanisms and contributing to longevity. *PLA2G7’s* identification as a critical regulatory gene is important to better understand the role of adipose tissue metabolism in regulating immune-metabolic effects. These data show that long-term calorie restriction (CR) improves health and extends life expectancy.^1^

Chronic 40% CR increases susceptibility to infections in mice, shortening their lifespan. In contrast, the Comprehensive Assessment of Long-Term Effects of Reducing Intake of Energy (CALERIE) analysis found that moderate CR in humans over 24 months did not reduce immune functions. 14% sustained CR was associated with improved adipose tissue metabolism, decreased inflammation, and reduced thymic lipoatrophy. Spadaro et al. discovered that low platelet activating factor acetyl hydrolase (*PLA2G7*) correlates with immune-metabolic consequences of CR and contributes to decreased inflammation and a longer lifespan in healthy mid-aged adults.^1^ PLA2G7 is a lipoprotein-associated calcium-independent phospholipase A2 involved in phospholipid catabolism during inflammatory and oxidative stress responses.

Divergent immunological responses were observed in animals with 40% CR, including greater susceptibility to viral and bacterial infections. This could be caused by reduced immunity, as in the context of CR, energetic resources are utilized for somatic cell maintenance rather than energy-intensive immune-modulatory functions. 40% CR causes a damaging stress response in rodents via glucocorticoids. In the CALERIE trial, modest sustained CR in healthy adults resulted in enhanced thymopoiesis and mobilization of intrathymic ectopic lipids. The aging of the thymus usually precedes the aging of other organs.^1^ The authors utilized these measures to define thymic function because thymic lipid accumulation and lower T cell production are linked to thymus aging. Total thymic volume increased in CALERIE study participants, as evidenced by an expansion of signal-joint T cell receptor excision circles (sjTREC) after 24 months of CR compared to baseline. sjTREC are markers for newly generated T cells in the thymus and are linked to good thymic function.

**Fig. 1:**
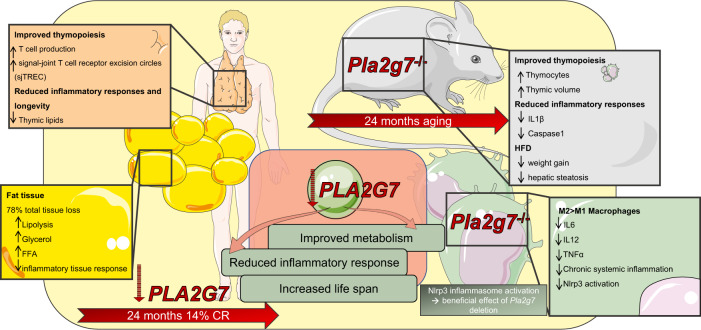
The Improvement of health and lower inflammation through Caloric Restriction (CR) via Pla2g7 downregulation. Improved thymopoiesis is found by enhanced T cell generation and greater levels of signal-joint T cell receptor excision circles (sjTREC) after a 14% CR for 24 months. Thymic lipid buildup after 24 months of CR was reduced in healthy mid-aged humans, which was used to estimate longevity. After CR, adipose tissue reduction accounted for 78% of overall weight loss. Increased lipolysis, as demonstrated by higher levels of glycerol and free fatty acids (FFA), helped to create an anti-inflammatory environment and improved adipose tissue metabolism. Downregulation of platelet activating factor acetyl hydrolase (PLA2G7) in humans experiencing CR is thought to be the origin of these effects, which mediates immune-metabolic effects that contribute to lower inflammation and a longer lifespan. The CRISPR Cas technique was used to generate Pla2g7^−/−^ knockout mice, which were protected from weight gain and steatosis when fed a high-fat diet (HFD). Thymocyte production and thymic volume were raised in these animals, highlighting the regulatory effects of *PLA2G7* in the human cohort. When compared to wildtype controls, the reduction of pro-inflammatory cytokines including Interleukin-1ß (IL1ß) and Caspase 1 contributed to the better phenotype of these mice after 24 months of age. Furthermore, these *Pla2g7*^−/−^ mice revealed a shift in macrophage polarization, with fewer pro-inflammatory M1 macrophages and more immunoregulatory M2 macrophages circulating in the bloodstream. In PLA2G7-depleted macrophages, total systemic inflammation was reduced, resulting in significantly reduced Interleukin 6 (IL6) and Interleukin 12 (IL12) levels as well as tumor markers. This figure was designed using smart.servier.com

For 14% CR in humans, a transformation of transcriptional reprogramming was identified in adipose tissue, e.g., lower anti-inflammatory responses, as evidenced by fewer lymphocytes and lower levels of pro-inflammatory cytokines. *PLA2G7* expression was shown to be reduced based on RNA sequencing data of total adipose tissue. *Pla2g7*-depleted mice were generated to functionally investigate this finding, these mice were protected from weight gain and hepatic steatosis when fed a high-fat diet. Increased adipose tissue lipolysis, as indicated by enhanced glycerol levels and free fatty acids, could be one of the mechanisms causing the improved phenotype in *Pla2g7* deficient animals (Fig. 1).

Recent results in autoimmune disease showed that abnormal lipid metabolism is triggering inflammation by causing oxidative stress. As a result, higher amounts of reactive oxygen species (ROS) are produced, driving macrophage activation.^2^ The scientists discovered decreased M1 macrophages and a lower expression of pro-inflammatory cytokines characteristic of the immunological phenotype of *Pla2g7* mice. However, CD206^+^ macrophages increased after *Pla2g7* deletion. M2 macrophages resolve inflammation and promote tissue repair. Spadaro et al. generated *Pla2g7* defective macrophages to unravel the role of Pla2g7 in the control of fundamental inflammatory pathways. These macrophages demonstrated a reduced activation of markers indicative of inflammasome activation in response to lipopolysaccharide (LPS) and ceramide stimulation. Ceramide-induced Nlrp3 activation has been linked to age-related thymic involution. *Pla2g7* knockout mice with a lifespan of 24 months had a larger thymic volume and a higher number of thymocytes. *Pla2g7* deficiency thus protected against age-related thymic involution. These findings in mice were consistent with the results in humans, which showed downregulation of *PLA2G7* during sustained CR.

Recent studies confirm the relevance of *Pla2g7* for shaping the immune phenotype in humans and mice.^2–4^ Lp-PLA2 (encoded by *Pla2g7*) levels were investigated as a possible biomarker in chronic obstructive pulmonary disease (COPD), a disease indicative for accelerated aging. In the context of chronic inflammation, Lp-PLA2 was increased. In the study by Spadaro and colleagues, however, there was a positive association between BMI and *Pla2g7* levels, indicating its regulation by body composition. This work highlights the role of *Pla2g7* in controlling the immune response by altering macrophage polarization by examining the inflammatory response in COPD patients.^4^

Lp-PLA2 has also been shown to have a significant function in modulating inflammatory macrophage (M1) polarization in animals with experimental autoimmune encephalitis (EAE). The effects of Lp-PLA2 on M1 polarization were mediated via oxidized LDL (oxLDL) and lysophosphatidylcholine (lysoPC), which could be reversed by an Lp-PLA2 inhibitor. *Pla2g7* inhibition is not only beneficial for autoimmune diseases like EAE, but it is also useful in reducing cardiac inflammation. Darapladip, an Lp-PLA2 inhibitor, reduced angiotensin II levels, which in turn reduced nlrp3 activation. These regulatory mechanisms are likely involved in the downregulation of macrophage-mediated fibroblast transformation.^3^

The downregulation of *Pla2g7* has been proposed by Spadaro et al. as a potential mechanism to achieve the favorable benefits of CR. In healthy people, 14% CR resulted in a considerable reduction of total weight (11,6%) and loss of visceral adipose tissue (VAT) (78%). Loss of VAT is believed to be one of the underlying processes explaining the favorable benefits of CR, given the previously described regulatory effects in adipose tissue metabolism. Even though sarcopenia is a significant risk factor for the progression of numerous diseases, the analysis of the CALERIE cohort revealed that lean tissue loss accounted for only 17.5% of total tissue loss, muscle strength was indeed preserved in the healthy adults who took part in the study.^5^

Spadaro and colleagues’ results underscore the importance of CR in lowering inflammation and validate its influence on life span in agreement with other publications.^2,4^ *Pla2g7*’s identification as a critical regulatory gene is a significant step forward in understanding the role of adipose tissue metabolism in regulating immune-metabolic effects. *Pla2g7*’s discovery as a key regulator of macrophage polarization should help to better understand inflammatory pathways in adipose tissue. These favorable data show that long-term CR improves health and extends the life expectancy. An unbalanced reduction in calorie intake, on the other hand, might significantly increase morbidity and death due to muscle loss and the breakdown of energetically costly immune-modulatory processes. This is evident in patients suffering from anorexia. As a result, aiming for a moderate CR is critical. Spadaro et al results stress further research in determining cut-off values for optimal CR objectives.
